# Supplementary material for: Time- and Dose-Dependent Effects of Dietary Deoxynivalenol (DON) in Rainbow Trout (Oncorhynchus mykiss) at Organism and Tissue Level
Source: Toxins (Basel). 2022 Nov 20;14(11):810. doi: 10.3390/toxins14110810 (PMC9697072; doi:10.3390/toxins14110810)
Supplement: Supplementary file 1 [file toxins-14-00810-s001.zip › toxins-2008302-Figure S1.pdf]

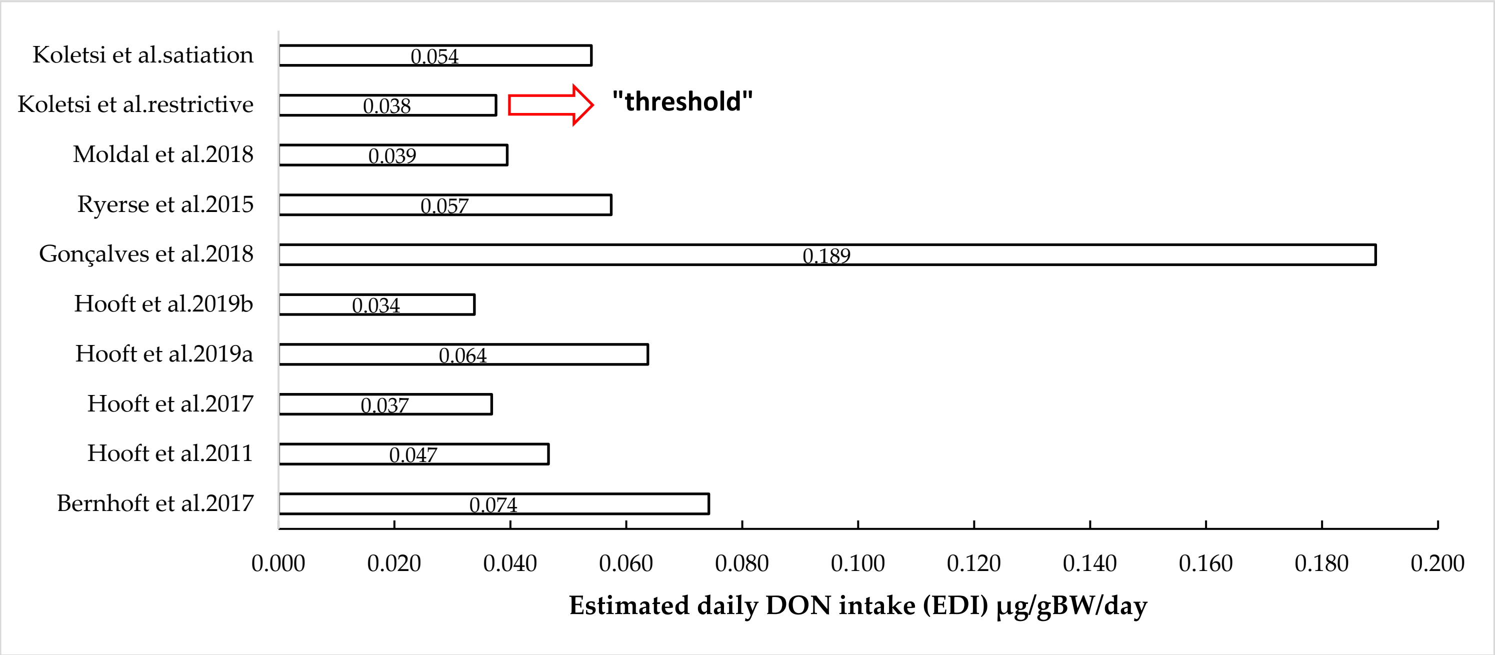

Figure S1. Comparative overview of the estimated daily DON intake (EDI) in salmonids studies, as it was calculated by using the highest DON dose that applied in each experiment.
In our study, the highest applied dose was the PD2=1600 μg/kg.
EDI (μg/gBW/day) = DON concentration in the feed* mean daily feed intake / average BW
