# Supplementary material for: Time- and Dose-Dependent Effects of Dietary Deoxynivalenol (DON) in Rainbow Trout (Oncorhynchus mykiss) at Organism and Tissue Level
Source: Toxins (Basel). 2022 Nov 20;14(11):810. doi: 10.3390/toxins14110810 (PMC9697072; doi:10.3390/toxins14110810)
Supplement: Supplementary file 1 [file toxins-14-00810-s001.zip › toxins-2008302-Figure S2.pdf]

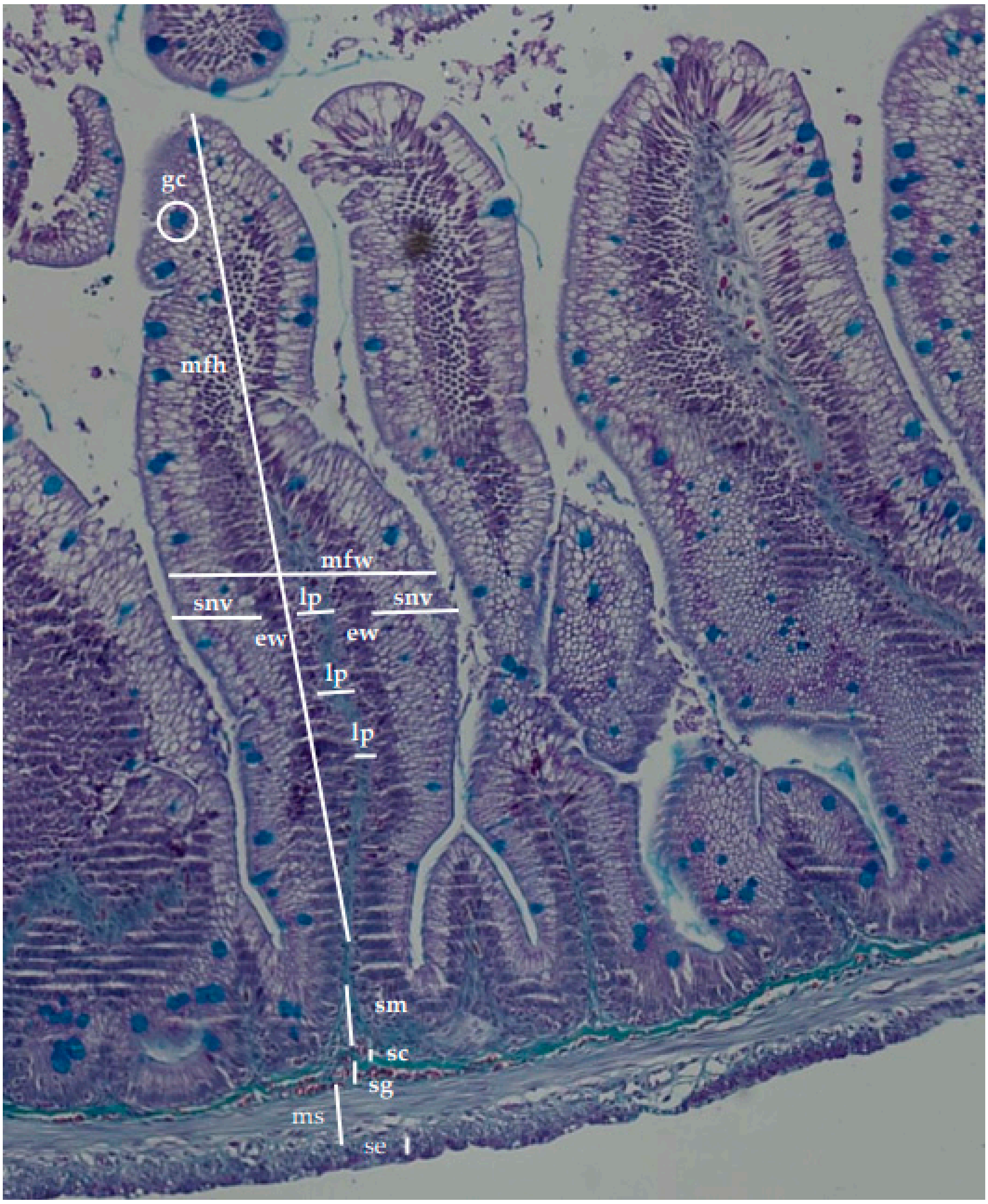

Figure S2. Parameters in the gastrointestinal tract used for the quantitative histopathological assessment: **se**; serosa, **ms**; muscularis, **sg**; stratum granulosum height, **sc**; stratum compactum height, **sm**; thickness of sub-epithelium mucosa, **lm**; lamina propria width, **ew**; enterocytes width, **snv**; supranuclear vacuoles width, **mfw**; mucosal fold width, **mfh**; mucosal fold height, **gc**; goblet cell.
