# Supplementary material for: Time- and Dose-Dependent Effects of Dietary Deoxynivalenol (DON) in Rainbow Trout (Oncorhynchus mykiss) at Organism and Tissue Level
Source: Toxins (Basel). 2022 Nov 20;14(11):810. doi: 10.3390/toxins14110810 (PMC9697072; doi:10.3390/toxins14110810)
Supplement: Supplementary file 1 [file toxins-14-00810-s001.zip › toxins-2008302-Table S1.pdf]

**Table S1**. Least square means and SEM of histological parameters in pyloric caeca, midgut and hindgut of rainbow trout after feeding restrictively for 6 days (week 1) and 40 days (week 6) and *ad libitum* for 15 days (week 8) the experimental diets; control (CON) , natural (ND2) and pure DON high (PD2).

| | | **Experimental diets** | | | | **p-value** | | | **$R^2$** |
|---|---|---|---|---|---|---|---|---|---|
| | **Week** | **CON** | **ND2** | **PD2** | SEM | diet | time | diet*time | |
| **Thickness of sub-epithelium mucosa (µm)** | | | | | | | | | |
| Pyloric | 1 | 47.28 | 57.95 | 47.85 | | | | | |
| | 6 | 46.74 | 49.59 | 59.06 | 5.03 | NS | NS | NS | 0.21 |
| | 8 | 62.53 | 57.29 | 53.32 | | | | | |
| Midgut | 1 | 42.38 | 54.23 | 46.65 | | | | | |
| | 6 | 48.03 | 53.92 | 53.39 | 4.94 | NS | NS | NS | 0.16 |
| | 8 | 52.63 | 54.98 | 57.95 | | | | | |
| Hindgut | 1 | $47.67^{ab}$ | $54.54^{ab}$ | $47.10^{ab}$ | | | | | |
| | 6 | $44.65^{a}$ | $52.91^{ab}$ | $54.61^{ab}$ | 4.15 | NS | * | NS | 0.33 |
| | 8 | $64.07^{b}$ | $62.41^{ab}$ | $51.58^{ab}$ | | | | | |
| **Mucosal fold width (µm)** | | | | | | | | | |
| Pyloric$^{\#}$ | 1 | $131.90^{ab}$ | $128.91^{ab}$ | $108.39^{a}$ | | | | | |
| | 6 | $125.70^{a}$ | $132.19^{ab}$ | $133.99^{ab}$ | 8.64 | NS | *** | NS | 0.59 |
| | 8 | $177.07^{c}$ | $168.87^{bc}$ | $166.30^{bc}$ | | | | | |
| Midgut | 1 | $130.85^{ab}$ | $124.97^{ab}$ | $102.18^{a}$ | | | | | |
| | 6 | $122.61^{ab}$ | $113.61^{ab}$ | $125.37^{ab}$ | 8.77 | NS | *** | NS | 0.40 |
| | 8 | $142.72^{b}$ | $149.37^{b}$ | $151.10^{b}$ | | | | | |
| Hindgut$^{\#}$ | 1 | $160.52^{ab}$ | $150.16^{ab}$ | $128.18^{a}$ | | | | | |
| | 6 | $141.14^{ab}$ | $135.46^{ab}$ | $153.73^{ab}$ | 7.44 | NS | * | ** | 0.39 |
| | 8 | $147.22^{ab}$ | $164.93^{b}$ | $166.87^{b}$ | | | | | |
| **Mucosal fold height (µm)** | | | | | | | | | |
| Pyloric | 1 | $387.53^{a}$ | $500.03^{ab}$ | $437.80^{a}$ | | | | | |
| | 6 | $397.15^{a}$ | $430.75^{a}$ | $547.48^{ab}$ | 50.86 | ** | NS | ** | 0.44 |
| | 8 | $387.55^{a}$ | $705.57^{b}$ | $446.95^{a}$ | | | | | |
| Midgut | 1 | $338.43^{a}$ | $364.76^{ab}$ | $382.07^{ab}$ | | | | | |
| | 6 | $355.31^{ab}$ | $413.47^{ab}$ | $382.66^{ab}$ | 42.76 | NS | *** | NS | 0.40 |
| | 8 | $517.46^{ab}$ | $514.63^{ab}$ | $540.93^{b}$ | | | | | |
| Hindgut | 1 | 347.96a | $396.25^{ab}$ | $323.17^{a}$ | | | | | |
| | 6 | $442.54^{ab}$ | $567.28^{bc}$ | $508.90^{abc}$ | 53.15 | NS | *** | NS | 0.48 |
| | 8 | $665.25^{c}$ | $585.07^{bc}$ | $534.26^{abc}$ | | | | | |
| **Lamina propria width average (µm)** | | | | | | | | | |
| Pyloric$^{\#}$ | 1 | $15.82^{ab}$ | $13.60^{a}$ | $13.02^{a}$ | | | | | |
| | 6 | $14.70^{ab}$ | $13.03^{a}$ | $12.69^{a}$ | 1.33 | NS | *** | NS | 0.60 |
| | 8 | $19.96^{b}$ | $22.81^{b}$ | $20.21^{b}$ | | | | | |
| Midgut | 1 | $14.16^{ab}$ | $16.50^{ab}$ | $12.11^{a}$ | | | | | |
| | 6 | $15.34^{ab}$ | $12.84^{a}$ | $17.97^{ab}$ | 1.75 | NS | *** | NS | 0.41 |
| | 8 | $18.71^{ab}$ | $20.53^{b}$ | $21.49^{b}$ | | | | | |
| Hindgut$^{\#}$ | 1 | $17.40^{ab}$ | $13.55^{a}$ | $12.32^{a}$ | | | | | |
| | 6 | $12.88^{a}$ | $13.29^{a}$ | $13.47^{a}$ | 1.66 | NS | *** | NS | 0.57 |
| | 8 | $21.30^{b}$ | $20.28^{b}$ | $23.68^{b}$ | | | | | |
| **Stratum granulosum width (µm)** | | | | | | | | | |
| Pyloric | 1 | $16.86^{a}$ | $20.98^{ab}$ | $21.53^{ab}$ | | | | | |
| | 6 | $21.58^{ab}$ | $24.05^{ab}$ | $29.99^{ab}$ | 3.05 | NS | ** | NS | 0.32 |

| | | | | | | | | | |
|---|---|---|---|---|---|---|---|---|---|
| | 8 | 33.13$^{b}$ | 25.67$^{ab}$ | 24.44$^{ab}$ | | | | | |
| Midgut | 1 | 21.86$^{a}$ | 28.41$^{ab}$ | 25.97$^{ab}$ | | | | | |
| | 6 | 42.13$^{b}$ | 29.20$^{ab}$ | 46.46$^{b}$ | 4.27 | NS | *** | NS | 0.43 |
| | 8 | 43.05$^{b}$ | 33.71$^{ab}$ | 40.19$^{ab}$ | | | | | |
| Hindgut | 1 | 22.43$^{a}$ | 26.23$^{ab}$ | 24.53$^{ab}$ | | | | | |
| | 6 | 27.58$^{ab}$ | 23.28$^{a}$ | 28.41$^{ab}$ | 3.55 | NS | * | NS | 0.33 |
| | 8 | 34.58$^{ab}$ | 39.32$^{b}$ | 23.96$^{ab}$ | | | | | |
| **Enterocytes width (μm)** | | | | | | | | | |
| Pyloric$^{\#}$ | 1 | 62.13$^{a}$ | 64.25$^{ab}$ | 50.94$^{a}$ | | | | | |
| | 6 | 58.25$^{a}$ | 64.06$^{ab}$ | 67.61$^{ab}$ | 5.41 | NS | *** | NS | 0.53 |
| | 8 | 86.50$^{b}$ | 85.58$^{b}$ | 82.97$^{b}$ | | | | | |
| Midgut | 1 | 58.99 | 60.55 | 51.14 | | | | | |
| | 6 | 57.79 | 53.68 | 58.90 | 5.54 | NS | ** | NS | 0.28 |
| | 8 | 64.40 | 74.14 | 72.84 | | | | | |
| Hindgut$^{\#}$ | 1 | 54.91$^{ab}$ | 55.93$^{ab}$ | 44.96$^{a}$ | | | | | |
| | 6 | 45.07$^{a}$ | 38.95$^{a}$ | 44.45$^{ab}$ | 3.82 | NS | *** | * | 0.43 |
| | 8 | 52.57$^{ab}$ | 54.01$^{ab}$ | 62.51$^{b}$ | | | | | |
| **Muscularis width (μm)** | | | | | | | | | |
| Pyloric | 1 | 28.23 | 35.66 | 28.66 | | | | | |
| | 6 | 37.44 | 46.60 | 43.51 | 7.27 | NS | NS | NS | 0.19 |
| | 8 | 52.74 | 39.04 | 38.88 | | | | | |
| Midgut | 1 | 38.92$^{a}$ | 51.66$^{ab}$ | 42.03$^{ab}$ | | | | | |
| | 6 | 81.20$^{ab}$ | 59.34$^{ab}$ | 79.08$^{ab}$ | 10.61 | NS | *** | NS | 0.37 |
| | 8 | 67.17$^{ab}$ | 87.08$^{b}$ | 85.46$^{ab}$ | | | | | |
| Hindgut | 1 | 47.81$^{a}$ | 55.11$^{ab}$ | 42.25$^{a}$ | | | | | |
| | 6 | 59.67$^{ab}$ | 40.80$^{a}$ | 48.45$^{ab}$ | 15.37 | NS | * | NS | 0.32 |
| | 8 | 62.33$^{ab}$ | 119.13$^{b}$ | 48.38$^{a}$ | | | | | |
| **Goblet cells density (per μm fold height)** | | | | | | | | | |
| Pyloric | 1 | 0.055 | 0.042 | 0.035 | | | | | |
| | 6 | 0.046 | 0.043 | 0.040 | 0.008 | NS | NS | NS | 0.12 |
| | 8 | 0.042 | 0.055 | 0.046 | | | | | |
| Midgut | 1 | 0.076$^{ab}$ | 0.058$^{a}$ | 0.072$^{ab}$ | | | | | |
| | 6 | 0.10$^{b}$ | 0.083$^{ab}$ | 0.083$^{ab}$ | 0.009 | NS | *** | NS | 0.39 |
| | 8 | 0.06$^{a}$ | 0.066$^{ab}$ | 0.048$^{a}$ | | | | | |
| Hindgut | 1 | 0.059 | 0.047 | 0.048 | | | | | |
| | 6 | 0.058 | 0.037 | 0.040 | 0.009 | NS | NS | NS | 0.17 |
| | 8 | 0.035 | 0.054 | 0.034 | | | | | |

Standard error of means: SEM (n=52), Not significant: NS, $p \leq 0.05$: *, $p \leq 0.01$: **, $p \leq 0.001$: ***
Per parameter and gut part, different superscripts (a, b, c) show significant differences ($p \leq 0.05$) according to Tukeys' multiple comparison test between all combinations of diet and time ; $^{\#}$n=51
